# Coexpression of Tail Fiber and Tail Protein Genes of the Cyanophage PP Using a Synthetic Genomics Approach Enhances the Salt Tolerance of *Synechocystis* PCC 6803

**DOI:** 10.1128/spectrum.05009-22

**Published:** 2023-05-01

**Authors:** Yu Chen, Pingbo Ge, Tao Sun, Jia Feng, Guorui Li, Jiabao Zhang, Jianting Zhou, Jianlan Jiang

**Affiliations:** a School of Chemical Engineering and Technology, Tianjin University, Tianjin, China; b Key Laboratory of Systems Bioengineering, Tianjin University, Tianjin, China; c Center for Biosafety Research and Strategy, Tianjin University, Tianjin, China; d Frontier Science Center for Synthetic Biology, Tianjin University, Tianjin, China; University of Guelph College of Biological Science

**Keywords:** artificial cyanophage genome, cyanophage PP, transcriptomic analysis, synthetic biology

## Abstract

Cyanophages are viruses that specifically infect cyanobacteria and are capable of regulating the population densities and seasonal distributions of cyanobacteria. However, few studies have investigated the interactions between cyanophages and heterologous hosts, owing to the inability of cyanophages to infect heterologous cyanobacterial hosts. Here, a truncated artificial cyanophage genome, Syn-P4-8, was designed and assembled that contained 18 genes for viral coat assembly proteins but not genes related to host infection or DNA replication. Syn-P4-8 was transferred into the heterologous host *Synechocystis* sp. PCC 6803 by conjugation. The growth of strain CS-02 carrying Syn-P4-8 was significantly better than that of the control strain when grown in medium containing 5% NaCl. Only two cyanophage genes, encoding the tail protein (open reading frame 25 [ORF25]) and the tail fiber protein (ORF26), were transcribed in *Synechocystis* PCC 6803 grown in BG11 medium supplemented with 5% NaCl. However, expression of either ORF25 or ORF26 alone could not recover this phenotype. In addition, transcriptomic analysis revealed the presence of 334 differentially expressed genes in CS-02 compared to the control strain, corresponding to 151 downregulated and 183 upregulated genes that may affect cyanobacterial salt tolerances. In this study, synthetic biology methods were used to strengthen our understanding of the interactions between cyanophage genes and heterologous hosts.

**IMPORTANCE** We synthesized and assembled a truncated cyanophage genome called Syn-P4-8, containing 18 genes for viral coat assembly proteins, and transferred it into a nonhost strain, *Synechocystis* sp. PCC 6803, to investigate interactions between Syn-P4-8 and *Synechocystis* PCC 6803. We found that coexpression of tail fiber and tail protein genes enhanced the salt tolerance of *Synechocystis* PCC 6803.

## INTRODUCTION

The occurrence of cyanobacterial blooms has become increasingly frequent due to water eutrophication and climate warming (1). Cyanophages are viruses that can specifically infect and lyse cyanobacteria, thereby dynamically regulating cyanobacterial communities in natural waters (2, 3). Consequently, the cyanophage has considerable application potential for controlling blooms. However, the infectibility of cyanophages relies on specific binding of receptor binding proteins to host surface receptors (4, 5). A previous study showed that most specialist cyanophages could not attach to the surfaces of nonhost cyanobacterial cells, resulting in failed infections (6). Thus, cyanophage genomes cannot be injected into nonhost cyanobacterial cells, although few studies have evaluated the interactions between cyanophages and nonhost cyanobacteria.

The synthetic technology underlying the generation of artificial genomes is mature, but few studies have investigated the synthesis of full-length artificial bacteriophage genomes. Nevertheless, the artificial synthesis of ϕX174 phage (7) and bacteriophage T7 (8) has been realized. Moreover, phage genomes can be edited and modularized by homologous recombination systems of yeast. For example, enhancement of tail genes in the phages T7 and K11 resulted in the original hosts of the two phages changing accordingly (9).

**FIG 1:**
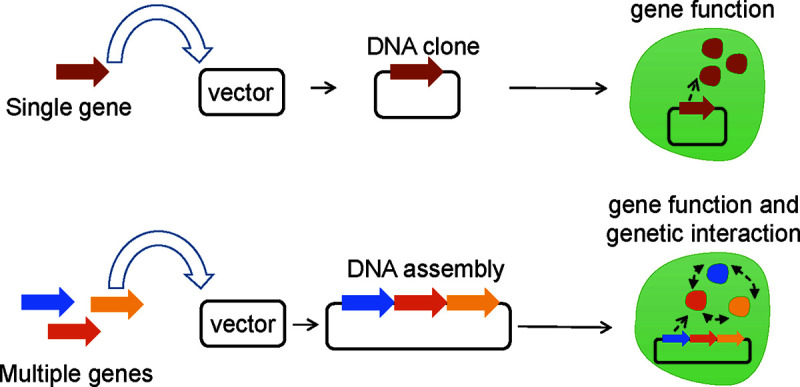
The synthetic genomic approach used to investigate interactions of cyanophages and hosts in the study.

The freshwater cyanophage PP (10) belongs to the *Podoviridae* family in the *Caudovirales* order and specifically infects *Plectonema boyanum* and *Phoridium foveolarum*. The PP genome comprises linear double-stranded DNA of length 42,480 bp and contains 222 bp of terminal repeats while encoding 41 open reading frames (ORFs) (11). Intriguingly, the genome can be divided into two completely different segments that differ in both orientation and function. Traditional gene overexpression of cyanophages usually targets a single gene (12). However, phage proteins often interact with and control other phage proteins, such as T7 RNA polymerase and T7 lysozyme (13). Thus, synthetic biological techniques can be used to synthesize genomes that contain multiple target genes so that it is easier to screen useful phenotypes generated by genetic interactions (Fig. 1).

In this study, synthetic biology approaches were used to synthesize and assemble viral coat assembly genes of a cyanophage, followed by transfer to *Synechocystis* sp. PCC 6803 to assess the effects of viral coat assembly genes on nonhost cyanobacteria (see Fig. S1 in the supplemental material). The results of this study will enhance the understanding of interactions between the cyanophage genome and heterologous hosts.

## RESULTS

### Synthesis and assembly of truncated Syn-P4-8.

**FIG 2:**
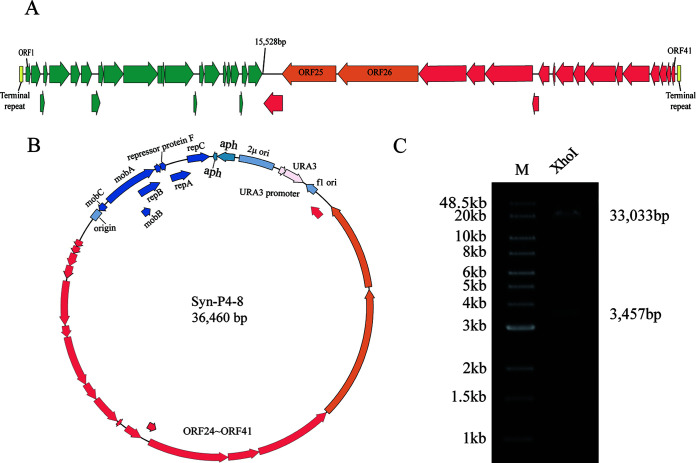
Synthesis and assembly of the cyanophage genome Syn-P4-8. (A) The cyanophage PP genome contained 41 ORFs (ORF1 to ORF41). (B) Genome map of the truncated Syn-P4-8 cyanobacteria genome. (C) Gel electrophoresis of the Syn-P4-8 enzymatic digestion. M, Quick Load 1-kb Extend DNA ladder.

Cyanophage PP can specifically infect *Plectonema boryanum* but not *Synechocystis* sp. PCC 6803. The cyanophage genome corresponds to GenBank accession number KF598865.1, is 41,850 bp long, and contains genes that include ORF1 to ORF41 (Fig. 2A; Table S4). The PP genome comprises 18 genes that are transcribed in the same direction as the viral coat assembly proteins. To validate whether the 18 genes interact with each other in heterologous hosts, the truncated genome was first synthesized and assembled from 60- to 90-bp oligonucleotides. Five DNA segments were first constructed with lengths of ~5.5 kbp from oligonucleotides by using multiple DNA assembly steps. The five DNA regions were then assembled using the transformation-associated recombination method in Saccharomyces cerevisiae using pCY0 as the vector (Fig. 2B; Fig. S2). The truncated cyanophage genome Syn-P4-8 was successfully constructed and verified using XhoI digestion (Fig. 2C). Sequencing confirmed the accuracy of the Syn-P4-8 sequence, with the exception of nine base mutations, of which three were synonymous (Table S2).

### Effects of Syn-P4-8 heterogenous expression on *Synechocystis* PCC 6803.

To assess the effects of Syn-P4-8 heterogeneous expression on *Synechocystis* PCC 6803, Syn-P-4-8 was transferred into *Synechocystis* PCC 6803 using triparental conjugative transfer, leading to the generation of strain CS-02 (Fig. S4). Concomitantly, a control cyanobacterial strain, CS-01, was constructed that carried an empty plasmid.

**FIG 3:**
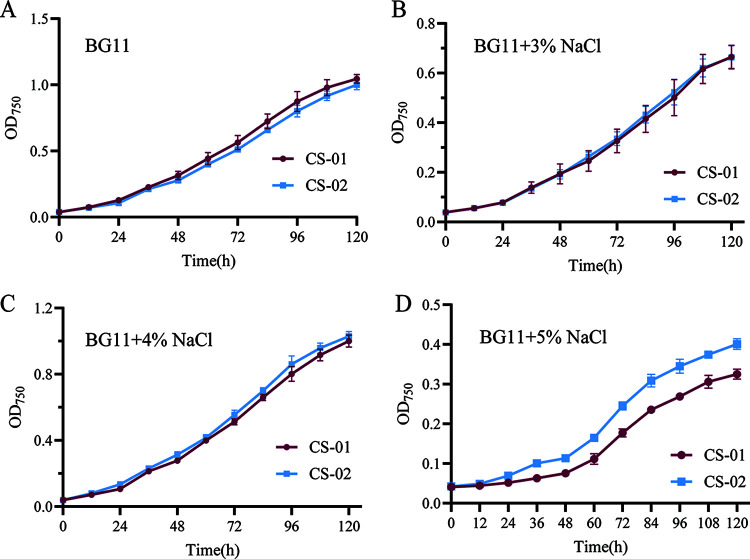
Effects of Syn-P4-8 on *Synechocystis* PCC 6803 cells under various culture conditions. Error bars represent standard deviations for three biological replicates for each sample. (A) Growth curves of CS-01 and CS-02 under normal BG11 medium conditions. (B) CS-01 and CS-02 growth curves during cultivation in 3% NaCl BG11 medium. (C) CS-01 and CS-02 growth curves when grown with 4% NaCl BG11 medium. (D) CS-01 and CS-02 growth curves during cultivation with 5% NaCl BG11 medium.

Strains CS-01 and CS-02 were cultured under various conditions to investigate the effects of Syn-P4-8 on the growth of the nonhost cyanobacterial strain *Synechocystis* PCC 6803 (Table S5). Under the same light intensity and temperature conditions [50 μmol/(m^2^·s) and 30°C, respectively], the growth curve of CS-02 was like that of CS-01 (Fig. 3). In BG11 medium supplemented with 5% NaCl, the CS-02 growth rate was higher than that of the control strain, CS-01. When the NaCl concentration was increased to 8%, the growth of the two strains was inhibited. In addition, when nitrogen-deficient BG11 medium was used for cultivation, both CS-01 and CS-02 cultures died within 2 days. During growth under 5% NaCl stress, the absorbances of CS-02 cells at 505, 625, and 680 nm were higher than those of CS-01. Thus, the salt tolerance of CS-02 was improved due to heterologous expression of Syn-P4-8.

### Coexpression of ORF25 and ORF26 increases the salt tolerance of *Synechocystis* PCC 6803.

**FIG 4:**
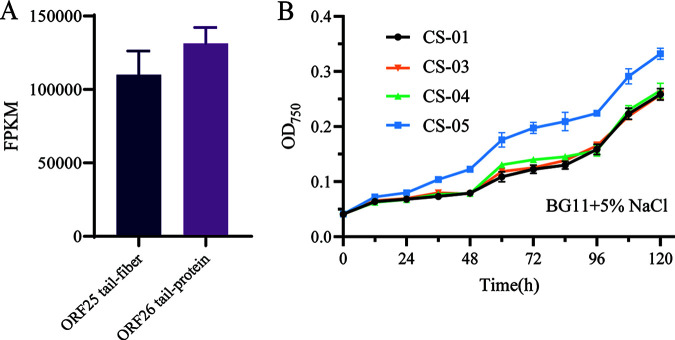
Growth differences resulting from the effects of ORF25 and ORF26 expression. Error bars indicate the standard deviations of the three biological replicates for each sample. (A) Expression of the cyanophage gene transcripts in the nonhost, *Synechocystis* PCC 6803. (B) Growth curves for CS-01, CS-03, CS-04, and CS-05 when grown in 5% NaCl BG11 medium.

To explore the mechanisms leading to increased salt tolerance, RNA sequencing (RNA-seq) was used to identify genes of Syn-P4-8 that were expressed in *Synechocystis* PCC 6803 cultured on BG11 medium supplemented with 5% NaCl. Among the 18 genes that encode the viral coat assembly proteins, only two (ORF25 and ORF26) were transcribed (Fig. 4A). ORF25 encodes the tail protein and ORF26 encodes the tail fiber protein. Thus, Syn-P4-8 was present in *Synechocystis* PCC 6803 cells, although most of its genes were not expressed. This result is consistent with previous studies that showed that nonhost cyanobacteria mitigate viral production by various intracellular interactions (14).

To further confirm that salt tolerance was related to heterologous expression of ORF25 and ORF26, an additional three recombinant plasmids (pCY03, pCY04, and pCY05) were constructed in S. cerevisiae using the pCY01 as a vector backbone. For expression of the gene of interest, the promoters of the target genes were predicted using the website http://www.softberry.com. All predicted promoters were included in the 200-bp sequence upstream of the each ORF’s start codon. (Fig. S3). During growth in 5% NaCl BG11 medium, CS-05, which encoded the two genes, grew faster than CS-01 after 20 h. The growth of both strains CS-03 and CS-04, which encoded only one ORF, was similar to that of the control (Fig. 4B). Similarly, the absorbances of CS-05 cells at 505, 625, and 680 nm were higher than those of CS-01. Thus, the expression of ORF25 and ORF26 alone cannot improve the salt tolerance of *Synechocystis* PCC 6803. Rather, the improved salt tolerance of the cyanobacteria resulted from the coexpression of the two genes.

### Transcriptomic analysis in BG11 medium with 5% NaCl.

**FIG 5:**
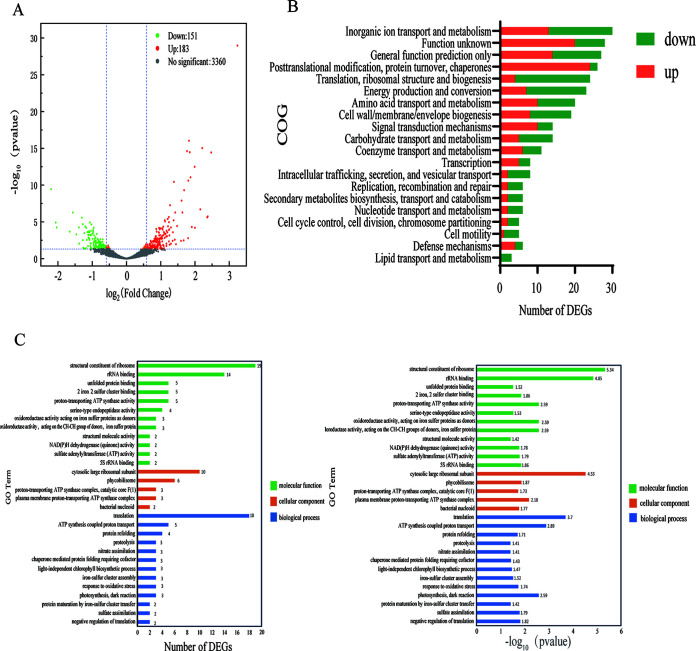
Response of *Synechocystis* PCC 6803 cells to heterologous expression of Syn-P4-8. (A) Volcano map of DEGs when grown in 5% NaCl BG11 medium. Red points indicate upregulation and green points indicate downregulation of genes. The *x* axis shows the log fold change in gene expression, while the *y* axis shows the statistical significance of gene expression differences. DEGs were identified based on the criteria of a fold change of >1.5 and a *P* value of <0.05. (B) Cluster of orthologous group family distribution of DEGs. (C) GO enrichment histogram. (Left) GO distribution of DEGs. The enriched GO terms are shown on the *y* axis and the *x* axis shows the number of DEGs for the term. Different colors distinguish different GO categories, including biological processes, cellular components, and molecular functions. (Right) Corresponding *P* values for GO terms shown on the left.

To evaluate the effects of SynP-4-8 on *Synechocystis* PCC 6803 at the transcriptional level, CS-02 and CS-01 were subjected to transcriptome analysis when cultivated in BG11 medium supplemented with 5% NaCl. The transcriptomic data were validated using quantitative real-time PCR (qRT-PCR), and the resulting correlation coefficients were >0.8, suggesting that the data were reliable (Fig. S5, Table S3). The fragments per kilobase per million mapped fragments (FPKM) metric was used to reflect the gene expression levels, and a total of 3,695 genes of *Synechocystis* PCC 6803 were expressed. Differentially expressed genes (DEGs) were identified based on gene expression fold changes of >1.5 and *Q* values (incorporating false discovery rate, adjusted *P* value [*P*_adj_]) of ≤0.05. A total of 3,695 genes were expressed by *Synechocystis* PCC 6803, of which 334 DEGs were identified, including 151 downregulated and 183 upregulated genes in CS-02 compared to CS-01, accounting for 9.04% of the total expressed genes (Fig. 5A). Some DEGs were identified with unknown functions, but these were associated with clusters of orthologous groups involved in inorganic ion transport and metabolism, posttranslational modification, protein turnover, chaperones, and translation functions, among others (Fig. 5B).

In particular, genes potentially involved in inorganic ion transport and metabolism were evaluated, and these comprised 13 upregulated and 20 downregulated genes. Most of these genes were related to thiometabolism in cyanobacterial cells. During salt stress, photorespiration and sulfur metabolism interact, leading to regulation of betaine synthesis in cyanobacteria and the regulation of cellular osmotic pressure, thereby improving the salt tolerance (15). We hypothesized that the heterologous expression of Syn-P4-8 in *Synechocystis* PCC 6803 upregulated these pathways and affected the synthesis of thiometabolites and sulfur compounds, with both, consequently, directly or indirectly improving the salt tolerance of cyanobacterial cells and promoting cell growth. These dynamics may ultimately explain differences in the salt tolerance of cyanobacteria.

To more thoroughly evaluate the physiological effects of identified DEGs, 30 genes with particularly significant differential expression were evaluated and subdivided into six groups (Table S7). The subset of DEGs was classified using GO annotations, and the distributions of the GO terms were evaluated (Fig. 5C). The most significantly enriched GO terms primarily included structural constituents of ribosomes, cytosolic large ribosomal subunits, and translation. The expression of these genes is very likely related to changes in the salt tolerance of *Synechocystis* PCC 6803, necessitating their further exploration.

## DISCUSSION

The 18 ORFs of the cyanophage PP are only related to the coat assembly of the cyanophage, and the constructed Syn-P4-8 lacked genes for host infection and DNA replication. Further, no significant change was observed in the growth of CS-02 in BG11 liquid medium [50 μmol/(m^2^·s), 30°C] relative to the control strain. Although it is worth exploring whether the salt tolerance of *Synechocystis* PCC 6803 also changed, no phenotypic changes were observed.

Heterologous infection of artificial cyanophages can theoretically be realized by using synthetic biology methods. Indeed, PP was used as a template to synthesize full-length artificial bacteriophages containing all ORFs in this study. However, a segment from bp 223 to 16,487 of PP containing the first 23 ORFs may contain some genes that are toxic to Escherichia coli, rendering it impossible for the full-length artificial bacteriophage to complete conjugation with cyanobacteria cells, as mediated by E. coli. Thus, additional research cannot be conducted with PP. This result is consistent with a previous study showing that cyanophage A-4L contains a region from bp 351 to 15,930 that also comprises genes that are toxic to E. coli, leading to an inability to clone the cyanophage (16).

### Conclusion.

Using Saccharomyces cerevisiae as a chassis, a portion of the artificial cyanophage genome Syn-P4-8 was successfully assembled in this study. Syn-P4-8 was then transferred to the nonhost cyanobacterial strain *Synechocystis* PCC 6803 via triparental conjugative transfer. The heterologous expression of Syn-P4-8 was then investigated in a nonhost cyanobacterial strain using transcriptome analysis. In 5% NaCl BG11 medium, CS-02 cells grew faster than CS-01 cells and transcriptional analysis suggested that this growth difference could be related to the joint action of ORF25 (tail fiber) and ORF26 (tail protein) proteins, thereby providing a basis for investigating the interactions between cyanophages and nonhost cyanobacteria.

## MATERIALS AND METHODS

### Strains and plasmids.

The strains and plasmids used in this study are shown in Table S1. Competent E. coli DH10B (NEB 10-beta electrocompetent) cells used in this study were purchased from Biomed, China. E. coli strains were cultured in LB medium at 37°C with antibiotics (50 μg/mL kanamycin), when applicable. Wild-type Saccharomyces cerevisiae BY4741 was grown in yeast extract-peptose-dextrose (YPD) medium at 30°C. Yeast transformants were selected and cultured on SC-Ura medium.

*Synechocystis* strains were cultured under normal conditions at 30°C, with a light density of 50 μmol/(m^2^·s). Wild-type *Synechocystis* PCC 6803 (ATCC 27184) (17) was grown in BG11 medium, and genetically constructed *Synechocystis* strains were cultured in BG11 medium supplemented with 25 μg/mL kanamycin (18). Fresh seed liquid was inoculated into 20 mL of BG11 medium supplemented with 25 μg/mL kanamycin to observe the growth of *Synechocystis* PCC 6803 strains under different culture conditions. The culture optical density (OD) at 750 nm and the full absorption spectra of liquid cultures were measured using a Varioskan LUX multifunctional microplate reader (Thermo Fisher Scientific). The initial OD_750_ of liquid cultures was 0.04 and was measured every 12 h. The full absorption spectra of the cultures were measured at 72 h. *Synechocystis* growth curves were evaluated by culturing *Synechocystis* on BG11 medium following nitrogen removal and salt addition at concentrations of 3%, 4%, 5%, 6%, and 8%.

To verify the effects of the two genes expressed in 5% NaCl BG11 medium on phenotypes, we first obtained three target DNA fragments containing ORF25, ORF26, or ORF25 and ORF26 by PCR using the PP genome as the template. pCY01 was linearized by PCR using primers F-line-3 and R-line-3 (listed in Table S3). The three fragments were separately cotransformed with linearized pCY01 into S. cerevisiae. Three plasmids (pCY03, pCY04, and pCY05) were successfully constructed and electroporated into NEB 10-beta cells (Biomed, China). Then, we carried out restriction enzyme analysis using EcoRV and NarI (Fig. S3). The recombinant plasmids were validated with PCR using the primers shown in Table S3.

### Preparation of yeast competent cells.

S. cerevisiae BY4741 was grown in 5 mL YPD at 30°C for 24 h. After overnight culture of yeast cells, they were added to 5 mL fresh medium and harvested once an OD_600_ of ~0.5 was reached. Yeast competent cells were prepared with 0.1 M LiOAc and maintained on ice (19).

### Construction of a truncated cyanophage genome and plasmid.

The truncated cyanophage fragments were synthesized using an artificial oligonucleotide synthesis strategy. The several DNA fragments (Table S1) were transferred into S. cerevisiae BY4741 cells by lithium acetate transformation for *in vivo* homologous recombination to construct corresponding plasmids and a truncated cyanophage genome. Each DNA fragment that we used contained 50 to 300 bp of homologous arms at both ends. The transformation system was prepared using the LiAc-SS carrier DNA-polyethylene glycol method (20). The cells were then spread onto corresponding solid nutrient-deficient media and cultured at 30°C for 2 days to identify positive clones.

### Transformation of *Synechocystis* PCC 6803.

Syn-P4-8 is a large DNA segment (>20 kbp) that was consequently diverted by triparental conjugative transfer (21) using the combined effects of a conjugative plasmid (pRL443) and the helper plasmid (pRL623) (22). Electroporation of the target plasmid Syn-P4-8 was conducted into NEB 10-beta cells (Biomed, China) as the donor strain. The target plasmid of the donor strain was diverted to *Synechocystis* PCC 6803 by triparental conjugative transfer. Briefly, 2 mL of log-phase E. coli cells containing the target plasmid and 2 mL of log-phase E. coli HB101 cells containing pRL443 and pRL623 were washed three times with LB medium without antibiotics. Then, 100 μL was resuspended in LB medium, mixed, and incubated at 37°C for 30 min. Then, 10 mL of log-phase *Synechocystis* PCC 6803 cells was centrifuged to 0.2 mL, which was added into the E. coli suspension, followed by evenly mixing. The mixture was then incubated at 30°C for 1 h and inoculated on BG11 plus 5% LB (vol/vol) agar plates, with a nitrocellulose membrane with pore diameter of 0.45 μm, at 30°C for 24 h under 50 μmol/(m^2^·s) light. The membrane was then transferred to a medium containing 25 μg/mL kanamycin and cultured on a BG11 agar plate for 3 to 5 days to screen stable transformants (23). Cyanobacterial transformants were verified by colony PCR (primers are shown in Table S3) and subjected to Sanger sequencing.

### Transcriptomic analysis.

Transcriptomic analysis was performed for *Synechocystis* strains cultured for 72 h with 5% NaCl stress to evaluate the heterologous expression of Syn-P4-8 in *Synechocystis* PCC 6803 and its effects on heterologous hosts. RNA-seq (24) was used for transcriptomic analysis at the Genewiz Sequencing Center (Suzhou, China). Three parallel replicates were included for each sample. The htseq software (v.0.6.1) was used to evaluate gene expression levels, based on the FPKM metric (25). Deseq2 (v.1.6.3) within the Bioconductor software program was then used to identify DEGs, based on comparisons of experimental and control groups. A total of 334 DEGs were identified with fold changes of >1.5 and a *P* value of <0.05.

### qRT-PCR.

Cyanobacterial cultures in liquid medium were incubated for 72 h, followed by centrifugation, immediate extraction of RNA, and freezing with liquid nitrogen. The RNA from each sample was quantified and used to synthesize cDNA using the Hiscript II Q RT Supermix for qPCR (+gDNA wiper). The obtained cDNA was diluted 100-fold and then used as the template for qRT-PCR. The qRT-PCR mixtures were prepared with the ChamQ Universal SYBR qPCR master mix (Vazyme, Nanjing, China) according to the manufacturer’s instructions. The qRT-PCRs were then performed using the StepOne real-time PCR system (Applied Biosystems, CA, USA). The *rnpB* gene encoding RNase P subunit B was used as a housekeeping gene. Three technical replicates were used for each sample. The qRT-PCR data were analyzed using the StepOne software program (Applied Biosystems) and the threshold cycle (2^−ΔΔ^*^CT^*) method (26).

Sixteen genes were selected to assess the reliability of RNA-seq expression data with qRT-PCR, using the primers shown in Table S6. Three technical replicates were used for each sample. Correlations between RNA-seq and qRT-PCR were assessed via Pearson correlation coefficients using the Excel software program. Correlation coefficients of >0.8 were used to identify reliable RNA-seq expression data.
