# Supplementary material for: Coexpression of Tail Fiber and Tail Protein Genes of the Cyanophage PP Using a Synthetic Genomics Approach Enhances the Salt Tolerance of Synechocystis PCC 6803
Source: Microbiol Spectr. 2023 May 1;11(3):e05009-22. doi: 10.1128/spectrum.05009-22 (PMC10269589; doi:10.1128/spectrum.05009-22)
Supplement: Supplemental file 1 — Supplemental material. Download spectrum.05009-22-s0001.pdf, PDF file, 0.7 MB [file spectrum.05009-22-s0001.pdf]

Table S1 Strains and plasmids used in this study

Name	Characteristics	Source
**strains**		
E. coli TOP10 competent cells	*F-mcrAΔ(mrr-hsdRMS-mcrBC)φ80lacZΔM15ΔlacX74recA1araΔ139Δ(ara-leu)7697galUgalKrpsL(strR)endA1nupG*	Biomed, China
NEB 10-beta Electrocompetent cells	*araD139△(ara,leu)7697 fhuA lacX74 galK16 galE15 mcrA80d(lacZM15)recA1 relA1 endA1 nupG rpsL rph spoT1△(mrrhsdRMS-mcrBC)*	Biomed, China
BY4741	*MATa his3Δ1 leu2Δ0 met15Δ0 ura3Δ0*	(16)
Synechocystis PCC 6803	Wide-type *Synechocystis* sp. PCC 6803 GT-G	ATCC 27184
CS-01	Syn6803 harboring the plasmid pJA	This study
CS-02	Syn6803 harboring genome Syn-P4-8	This study
CS-03	Syn6803 harboring the plasmid pCY03	This study
CS-04	Syn6803 harboring the plasmid pCY04	This study
CS-05	Syn6803 harboring the plasmid pCY05	This study
**plasmids**		
pRL623	*CmR*	(19)
pRL443	*AmpR*	(19)
pJA	*KanR*，*E. coli*-cyanobacteria shuttle vector	This laboratory
Syn-P4-8	pJA harboring the cassette P4-P8	This study
pCY0	pJA harboring the *URA3*	This study
pCY01	pJA harboring the *URA3*	This study
pCY03	pCY01 harboring the ORF25 of PP	This study
pCY04	pCY01 harboring the ORF26 of PP	This study
pCY05	pCY01 harboring the ORF26 and ORF26 of PP	This study

Table S2 Sequencing results of assembled artificial cyanophage genomes Syn-P4-8

Locations	Base mutation	Codon mutation
511bp	A-G	synonymous mutation
546bp	C-A	missense mutation
5134bp	C-G	missense mutation
5455bp	G-T	missense mutation
5658bp	C-G	synonymous mutation
15,849bp	G-T	synonymous mutation
1206bp	C-A	missense mutation
15,849bp	G-T	missense mutation
21,451bp	G-T	missense mutation

Table S3 All the primers used in this study.

Primers	Sequences (5'-3')
**Primers involved in the assembly for Syn-P4-8**	
F-P4	CCCGTAAAGGAAGGCGAAGC
R-P4	CCCACTATATGGTCAACCTACAGGC
F-P5	GGTACTAGTGTTGGGTGAACATCTAG
R-P5	GAGGTGATCTCAAGCTTACGTGAC
F-P6	CTCTAAAAGGGGGTGTAGTACCTCC
R-P6	CTTTAGTAACGTGTTTTCCGCTGC
F-P7	GGTTGTCGAGTTATACGAGTACGTAG
R-P7	CCAGATGAAGACAACCTACTTGTGTC
F-P8	GGTCATAGAAGCAGTCAAACATATCCAG
R-P8	ACACATACTATAAGCACACTGTGGATG
F-liner-1	CACAACGTGGCTTTGTTGAATAAATCGAACTTTTGCTGAGTTG
	AAGGATCAGCTCGAGTTTCCCCGAAAAGTGCCACCTG
R-liner-1	GTTAACTAACTATGCGTTATCTGCTTCGCCTTCCTTTACGGGGA
	ACCCGCGGCCGCCGGGCTGCAGGAATTCGATATC
F-liner-2	GTGAGCAAAAGGCCAGCAAAAGGCCAGGAACCGTAAAAAGG
	CCGCGTTGCTGGCTCACTCAAAGGCGGTAATCAATTG
R-liner-2	GTTCTACAAAATGAAGCACAGATGCTTCGTTCAGGTGGCACTT
	TTCGGGGAAACTCGAGCTGATCCTTCAACTCAGC
**Primers involved in the validation for Syn-P4-8**	
F-test- Syn-P4-8-1	CTTCCTCGCCAGTTCGCTC
R-test- Syn-P4-8-1	AAGCACCACAAAACGACCC
F-test- Syn-P4-8-2	AGGGTAACAGGTTCTCGCC
R-test- Syn-P4-8-2	AACGGTATCCCGATCCCTG
F-test- Syn-P4-8-3	CGGGTTCATCAGTCGTTCG
R-test- Syn-P4-8-3	GGTAAACAACCCCGCTGAG
F-test- Syn-P4-8-4	GACTGGTCAGCTTTGTTCACG
R-test- Syn-P4-8-4	GAAAGAAGAGGTCAAGGAGCG
F-test- Syn-P4-8-5	CCGATGACAAACGGACGAC
R-test- Syn-P4-8-5	GCTTGGGCATCTTACCTCAG
F-test- Syn-P4-8-6	CTTCCTCGCCAGTTCGCTC
R-test- Syn-P4-8-6	AAGCACCACAAAACGACCC
F-test- Syn-P4-8-7	AGGGTAACAGGTTCTCGCC
R-test- Syn-P4-8-7	AACGGTATCCCGATCCCTG
**Primers involved in the validation for pJA**	
F-pJA-1	GAATTACCCTCCACGTTGATTGTC
R-pJA-1	CAAGCTCGAAGAACAACGAGC
F-pJA-2	CGCATCAACCAAACCGTTATTC
R-pJA-2	CCGCTTACAGACAAGCTGTG
**Primers involved in the linearization for pCY01**	
F-line-3	GCTCACTCAAAGGCGGTAATCAATTG

R-line-3	GCGGCCGCTTGTTCATGTGTGTTCAAAAAC
**Primers involved in the validation for pCY03**	
F-test- pCY03-1	CACCATGAGTGACGACTGAATCC
R-test- pCY03-1	CTAGGTAAGGACAGTAACCCTGC
F-test- pCY03-2	GTCATGCGTACTTCATCAAACACG
R-test- pCY03-2	CACGTATCAAACCTACGGCTGTC
F-test- pCY03-3	GTAGCCTTAGCAGTAGCCTCATC
R-test- pCY03-3	GCAGGATAGAACATACTACCGTCAG
F-test- pCY03-4	GTGGGATGTTCAGTAGTTGCATACC
R-test- pCY03-4	CTTACAGACAAGCTGTGACCGTC
**Primers involved in the validation for pCY04**	
F-test- pCY04-1	CACCATGAGTGACGACTGAATCC
R-test- pCY04-1	GTCAGTGAGATCATCAGTGGTCG
F-test- pCY04-2	GTTAAAACGCTCGACACGTCTATC
R-test- pCY04-2	CTTACAGACAAGCTGTGACCGTC
**Primers involved in the validation for pCY05**	
F-test- pCY05-1	CACCATGAGTGACGACTGAATCC
R-test- pCY05-1	CGTATCAAACCTACGGCTGTCATC
F-test- pCY05-2	GTAGCCTTAGCAGTAGCCTCATC
R-test- pCY05-2	GCAGGATAGAACATACTACCGTCAG
F-test- pCY05-3	GTGGGATGTTCAGTAGTTGCATACC
R-test- pCY05-3	GACGAAAGGGCCTCGTGATAC
**Primers for qRT-PCR**	
F1-PCR	ACTCTGGTGGTCCTCTCCTC
R1-PCR	TCCACTTTGCCCGTAGCAAT
F2-PCR	GGGAAGAACTGGGGTGATGG
R2-PCR	TGAATACCCGTTCCTTGGGC
F3-PCR	AAGTACGGGGAAAACGTCGG
R3-PCR	CAAATGCTGAACCATCCCCG
F4-PCR	AACTATGGCCCAACTCGTGG
R4-PCR	AAGACTTAGCCCGCTTTCCC
F5-PCR	TACGGCGCTGAGTTGGATTT
R5-PCR	TTGATTGCGTTCTTGCAGCC
F6-PCR	CTCTGGCATCACTGTCACCA
R6-PCR	AAGCTCCAGGGCAGTCTTTG
F7-PCR	TTGGTGGTTCGGATACGGTG
R7-PCR	AACAGCGGGAGTACCTGTTG
F8-PCR	TTTCCTATCCTCTGGCGGGA
R8-PCR	ATCGGAGTGGAGGGTCGTAA
F9-PCR	CGGTATAGCGAATGGGGAGG
R9-PCR	TGGTATCGAAGCCGCTAACC
F10-PCR	ACGCCACGGTATTTGATTGC
R10-PCR	CGCCCAAATCAACAGTTCCC
F11-PCR	CCACCGCTTTGGTTTCACTG

R11-PCR	AATGGCTCCTCAGCACCTTC
F12-PCR	CCCGTTTAGCTTCTCCTCCC
R12-PCR	TCGCCCACCTTAGCAAAACA
F13-PCR	GAATCCAGGGGAACCCGTTT
R13-PCR	AACCTTGGCGTACCATAGCC
F14-PCR	TAGTTGGACTGCCGAAGCAA
R14-PCR	AGCTTGCTCGTAGACCGTTT
F15-PCR	CCGGTGTTTTGAAGCCAAGG
R15-PCR	ACTAAAATGGGCCGCCATGA
F16-PCR	ACATGCAAGTCGAACGGAGT
R16-PCR	TAATCAGACGCGAGCCCATC

Table S4 Information of all ORFs in PP genome

ORF	Predicted protein	location
ORF1	hypothetical protein	419-685 bp
ORF2	hypothetical protein	744-1334 bp
ORF3	hypothetical protein	1331-1567 bp
ORF4	hypothetical protein	1545-1748 bp
ORF5	hypothetical protein	1949-3202 bp
ORF6	hypothetical protein	3292-3837 bp
ORF7	hypothetical protein	3956-4624 bp
ORF8	hypothetical protein	4624-5133 bp
ORF9	hypothetical protein	5078-5371 bp
ORF10	hypothetical protein	5377-6636 bp
ORF11	DNA primase/helicase	6641-8815 bp
ORF12	hypothetical protein	8876-9076 bp
ORF13	hypothetical protein	9089-9295 bp
ORF14	DNA polymerase	9299-11167 bp
ORF15	hypothetical protein	11,149-11,304 bp
ORF16	hypothetical protein	11,497-11,742 bp
ORF17	hypothetical protein	11,826-12,788 bp
ORF18	hypothetical protein	13,017-13,193 bp
ORF19	hypothetical protein	13,272-13,514 bp
ORF20	ERF family protein	13,522-14,025 bp
ORF21	hypothetical protein	14,038-14,247 bp
ORF22	hypothetical protein	14,210-14,548 bp
ORF23	hypothetical protein	14,627-15,487 bp
ORF24	tail fiber	15,551-15,673 bp
ORF25	tail fiber	16,740-20,201 bp
ORF26	tail protein	20,299-25,437 bp
ORF27	endopeptidase	25,485-28,469 bp
ORF28	internal protein	28,471-29,661 bp
ORF29	tail tube B	29,665-32,748 bp
ORF30	recombination endonuclease VII	32,738-33,103 bp
ORF31	tail tube A	33,108-33,773 bp
ORF32	hypothetical protein	34,050-34,166 bp

ORF33	capsid protein	34,216-35,361 bp
ORF34	scaffolding protein	35,384-36,067 bp
ORF35	portal protein	36,072-38,027 bp
ORF36	DNA endonuclease	38,042-38,467 bp
ORF37	terminase	38,504-40,201 bp
ORF38	hypothetical protein	40,307-40,807 bp
ORF39	hypothetical protein	40,813-41,298 bp
ORF40	hypothetical protein	41,319-41,594 bp
ORF41	hypothetical protein	41,599-41,850 bp

Table S5 Culture conditions for phenotypic screening

Culture medium	Light intensity	Temperature
BG11 medium	50 μmol/(m^2·s)	30 ℃
BG11 medium +3%NaCl	50 μmol/(m^2·s)	30 ℃
BG11 medium +4%NaCl	50 μmol/(m^2·s)	30 ℃
BG11 medium +5%NaCl	50 μmol/(m^2·s)	30 ℃
BG11 medium +6%NaCl	50 μmol/(m^2·s)	30 ℃
BG11 medium +8%NaCl	50 μmol/(m^2·s)	30 ℃
nitrogen deficient BG11 medium	50 μmol/(m^2·s)	30 ℃

Table S6 Comparison of RT qPCR analysis with RNA-seq for selective transcripts

GeneName	Product	RT-qPCR ratio	RNA-seq ratio
SGL_RS06030	trypsin-like peptidase domain-containing protein	5.36	9.44
SGL_RS06045	efflux RND transporter periplasmic adaptor subunit	2.66	5.56
SGL_RS08225	DUF1232 domain-containing protein	2.00	5.18
SGL_RS16955	site-2 protease family protein	1.37	5.12
SGL_RS04110	hypothetical protein	1.99	4.62
SGL_RS07935	SpoIIE family protein phosphatase	0.70	0.21
SGL_RS11695	ABC transporter ATP-binding protein	0.48	0.23
SGL_RS16075	H(+)/Cl(-) exchange transporter ClcA	0.52	0.24
SGL_RS02885	PCP reductase family protein	1.01	0.31
SGL_RS17665	NAD(P)/FAD-dependent oxidoreductase	0.92	0.33
SGL_RS17190	hypothetical protein	0.62	0.99
SGL_RS09035	hypothetical protein	0.58	0.99
SGL_RS08170	MFS transporter	0.72	1.00
SGL_RS06735	BMC domain-containing protein	0.72	1.00
SGL_RS17540	DUF4278 domain-containing protein	0.72	1.01

Table S7 The DEGs with functional annotations under 5% NaCl stress

GeneName	$log_2(FC)$	*p*-value	Product
**Group 1：DEGs involved in sulfur metabolism**			
SGL_RS15255	0.86547	0.000479	Fe-S cluster assembly ATPase SufC
SGL_RS02980	0.96327	0.000718	peptide-methionine (R)-S-oxide reductase MsrB
SGL_RS09160	1.249157	2.47E-06	thioredoxin family protein
SGL_RS15260	0.679371	0.006436	Fe-S cluster assembly protein SufD
SGL_RS04455	0.793964	0.00217	Grx4 family monothiol glutaredoxin
**Group 2：DEGs involved in carbohydrate metabolism**			
SGL_RS06955	0.741609	0.006419	carbohydrate kinase
SGL_RS05290	0.968608	9.69E-05	NAD-dependent epimerase/dehydratase family protein
SGL_RS07215	0.741177	0.001937	type I glyceraldehyde-3-phosphate dehydrogenase
SGL_RS09150	0.725029	0.001741	acetolactate synthase large subunit
SGL_RS11955	0.715926	0.004217	aminomethyl-transferring glycine dehydrogenase
**Group 3 ：DEGs encoding in transporters**			
SGL_RS08385	0.95172	9.36E-06	MFS transporter
SGL_RS06020	0.829821	0.000125	sugar ABC transporter permease
SGL_RS08095	1.075314	7.38E-07	peptide ABC transporter substrate-binding protein
**Group 4：DEGs encoding in heat shock protein and signal transduction mechanisms**			
SGL_RS13680	1.990653	1.09E-15	chaperonin GroEL
SGL_RS06170	1.869704	3.36E-14	chaperonin GroEL
SGL_RS12750	1.609996	8.12E-11	molecular chaperone DnaK
SGL_RS11615	1.043377	1.64E-06	ATP-dependent chaperone ClpB
SGL_RS13375	0.984184	6.90E-05	ATP-dependent Clp protease ATP-binding subunit
SGL_RS18085	0.586984	0.008078	molecular chaperone HtpG
SGL_RS06165	1.806297	1.06E-13	co-chaperone GroES
SGL_RS15370	0.895438	0.006989	DnaJ domain-containing protein
**Group 5：DEGs involved in photosynthesis**			
SGL_RS11045	1.299848	1.05E-06	glutathione peroxidase
SGL_RS08615	0.952418	2.75E-05	glutathione peroxidase

**Group 6：DEGs involved in transcription and translation**			
SGL_RS02985	0.733192	0.003653	trypsin-like peptidase domain-containing protein
SGL_RS06030	3.238927	5.09E-33	trypsin-like peptidase domain-containing protein
SGL_RS09260	0.797089	0.000355	trypsin-like peptidase domain-containing protein
SGL_RS03485	0.8333	0.000233	translation initiation factor IF-3
SGL_RS14220	0.84748	4.12E-05	30S ribosomal protein S14
SGL_RS13145	0.695652	0.002821	30S ribosome-binding factor RbfA
SGL_RS05695	0.772338	0.002785	50S ribosomal protein L25

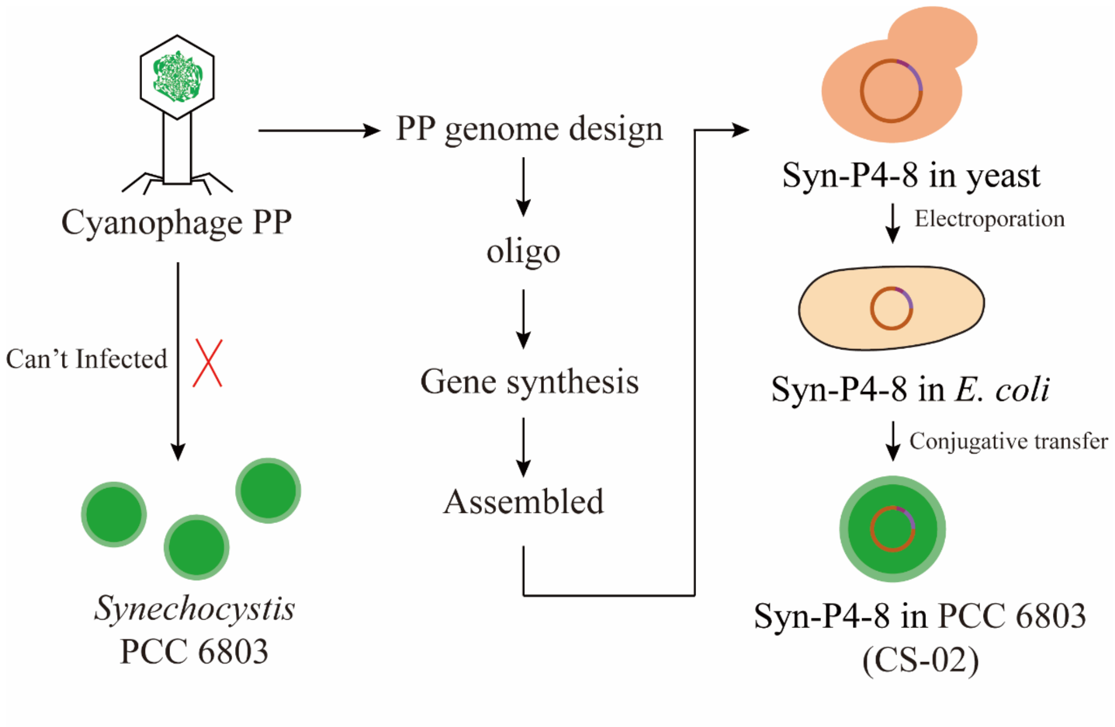

Fig. S1 Schematic diagram of research route in this study.

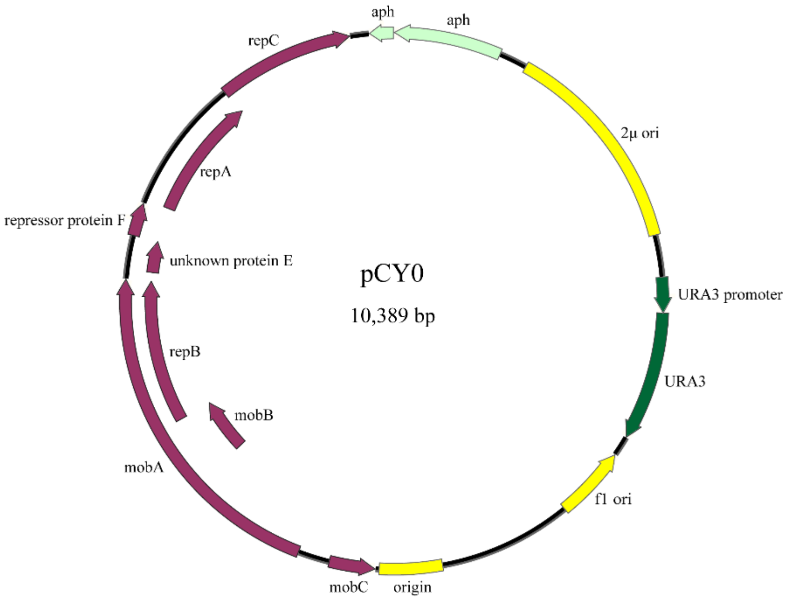

Fig. S2 Map of the pCY0

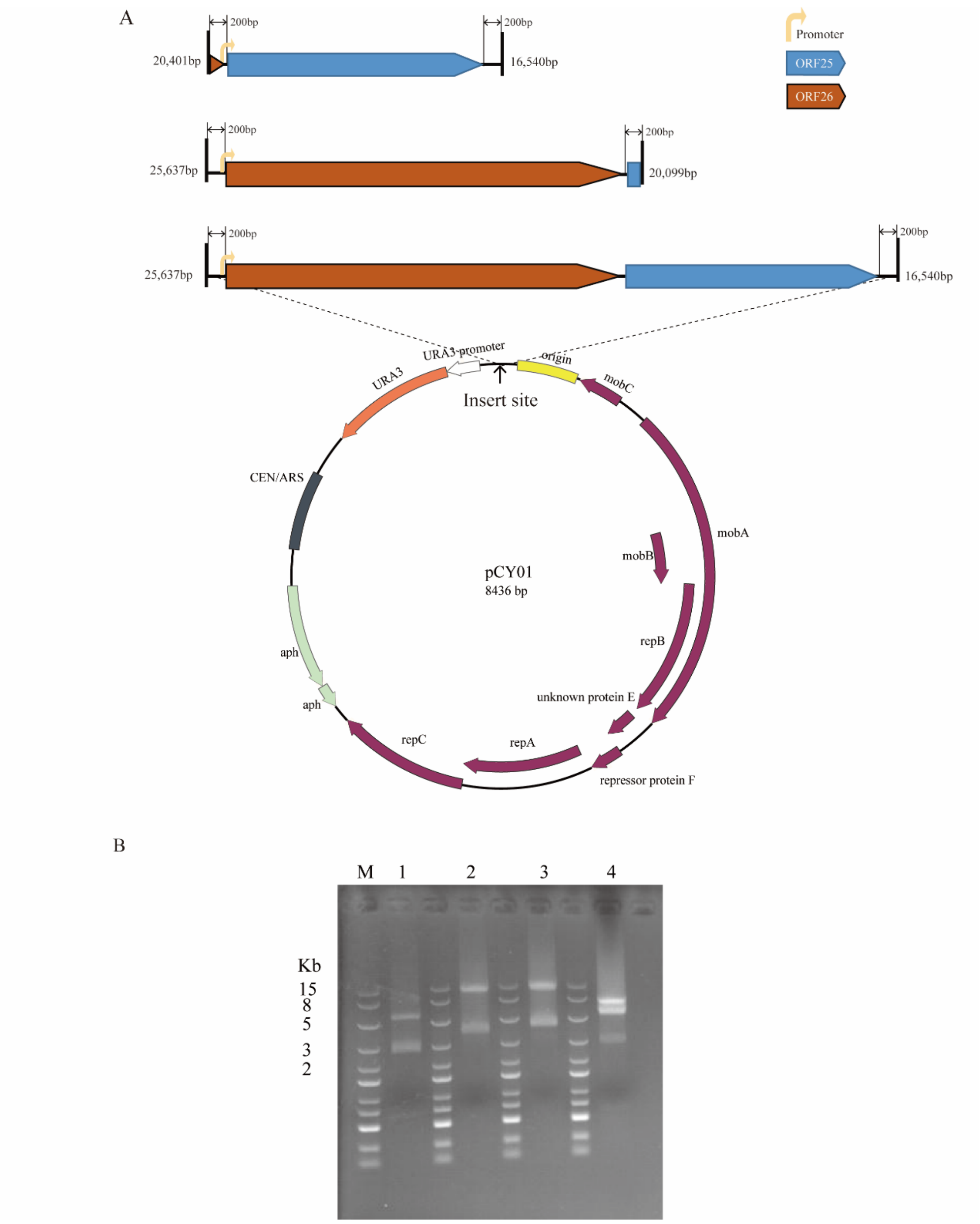

Fig.S3 Construction of pCY03, pCY04 and pCY05.

A: pCY01 was used as the vector. pCY03 containing genome region:16,540-20,401 bp, the location of the predicted promoters (-10 box: 20,289-20,294 bp, -35 box: 20,307-20,315 bp); pCY04 containing genome region:20,099-25,637 bp, the location of the predicted promoter (-10 box: 25,482-25,489 bp, -35 box: 25,506-25,511 bp); pCY05 containing genome region: 16,540-25,637 bp, the location of the predicted promoter (-10 box: 25,482-25,489 bp, -35 box: 25,506-25,511 bp).

B: Digestion analysis of pCY03, pCY04 and pCY05. M: 250 bp DNA Ladder. Lane 1: The plasmid pCY01 was digested by *EcoR* V (5657 bp, 2779 bp). Lane 2: The plasmid pCY03 was digested by *Nar* I (8753 bp, 3345 bp). Lane3: The plasmid pCY04 was digested by *Nar* I (10,430

bp, 3345 bp). Lane 4: The plasmid pCY05 is digested by *EcoR* V (7441 bp, 5657 bp, 3128 bp).

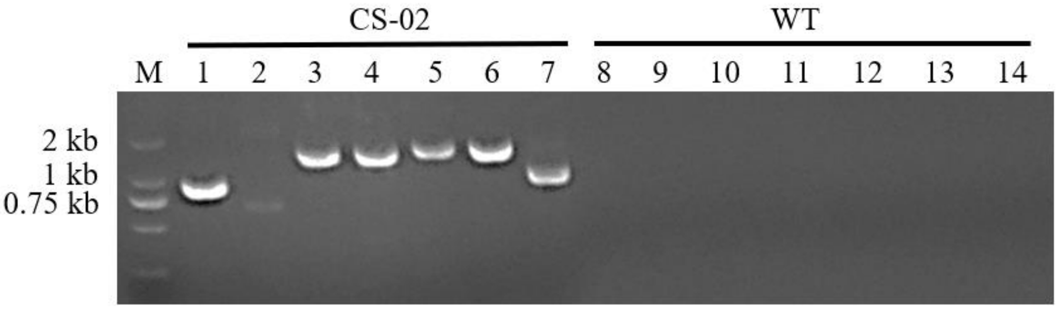

Fig. S4 Gel electrophoresis of PCR verification for CS-02.

CS-02: lane 1-7, Syn6803 harboring the genome Syn-P4-8; WT: lane 8-14, wild type Syn6803. The length of PCR products was 887 bp, 743 bp, 1393 bp, 1299 bp, 1525 bp, 1364 bp, 996 bp respectively. M: DL2000 DNA Marker.

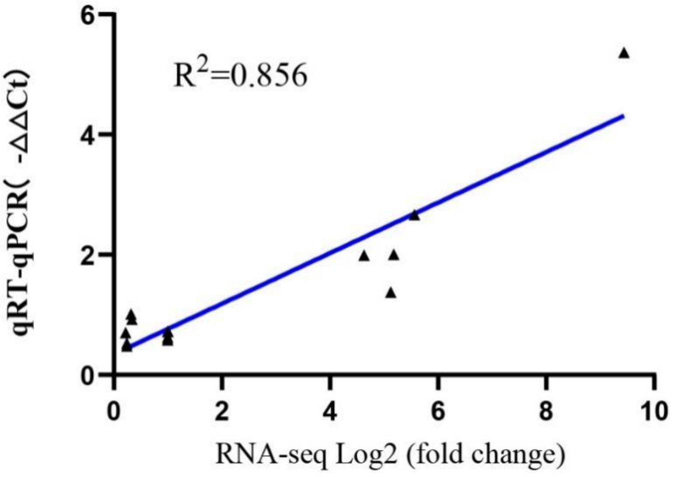

Fig. S5 Pearson correlation coefficient scatter plots.
